# *Bordetella pertussis* Whole Cell Immunization, Unlike Acellular Immunization, Mimics Naïve Infection by Driving Hematopoietic Stem and Progenitor Cell Expansion in Mice

**DOI:** 10.3389/fimmu.2018.02376

**Published:** 2018-10-18

**Authors:** Melinda E. Varney, Dylan T. Boehm, Katherine DeRoos, Evan S. Nowak, Ting Y. Wong, Emel Sen-Kilic, Shebly D. Bradford, Cody Elkins, Matthew S. Epperly, William T. Witt, Mariette Barbier, F. Heath Damron

**Affiliations:** ^1^Department of Microbiology, Immunology, and Cell Biology, West Virginia University School of Medicine, Morgantown, WV, United States; ^2^Vaccine Development Center at West Virginia University Health Sciences Center, Morgantown, WV, United States

**Keywords:** vaccination, *Bordetella pertussis*, respiratory pathogen, hematopoietic stem cell, immunobiology

## Abstract

Hematopoietic stem and progenitor cell (HSPC) compartments are altered to direct immune responses to infection. Their roles during immunization are not well-described. To elucidate mechanisms for waning immunity following immunization with acellular vaccines (ACVs) against *Bordetella pertussis* (*Bp*), we tested the hypothesis that immunization with *Bp* ACVs and whole cell vaccines (WCVs) differ in directing the HSPC characteristics and immune cell development patterns that ultimately contribute to the types and quantities of cells produced to fight infection. Our data demonstrate that compared to control and ACV-immunized CD-1 mice, immunization with an efficacious WCV drives expansion of hematopoietic multipotent progenitor cells (MPPs), increases circulating white blood cells (WBCs), and alters the size and composition of lymphoid organs. In addition to MPPs, common lymphoid progenitor (CLP) proportions increase in the bone marrow of WCV-immunized mice, while B220^+^ cell proportions decrease. Upon subsequent infection, increases in maturing B cell populations are striking in WCV-immunized mice. RNAseq analyses of HSPCs revealed that WCV and ACV-immunized mice vastly differ in developing VDJ gene segment diversity. Moreover, gene set enrichment analyses demonstrate WCV-immunized mice exhibit unique gene signatures that suggest roles for interferon (IFN) induced gene expression. Also observed in naïve infection, these IFN stimulated gene (ISG) signatures point toward roles in cell survival, cell cycle, autophagy, and antigen processing and presentation. Taken together, these findings underscore the impact of vaccine antigen and adjuvant content on skewing and/or priming HSPC populations for immune response.

## Introduction

Innate and adaptive immune cells originate from hematopoietic stem cells (HSCs). Over an organism's lifetime, long-term HSCs (LT-HSCs) self-renew and generate short-term HSCs (ST-HSCs). ST-HSCs give rise to myeloid and lymphoid progenitors that differentiate into immune cells (1). Hematopoietic stem and progenitor cell (HSPC) renewal, expansion, and differentiation are tightly regulated to produce and maintain sufficient blood cells (2). Cell–cell interactions, microenvironment contributions, cytokines, and gene signature changes prompt HSPC expansion (1, 3). During infection, this results from: (1) direct pathogen interaction or signals that push differentiation and/or mobilization or (2) a pull to replenish leukocytes required for immune surveillance and clearing infection (4, 5). The immune system critically relies on these events to produce correct quantities and types of cells required for response to pathogens and stress (6). At sites of infection, innate and adaptive arms of the immune system work synergistically to clear infection and/or prevent recurrence. Long-term immunity is maintained by antigen-specific memory B and T cells. Recent evidence suggests that innate immune cells also provide long-term immunity independently (7–9).

While HSPCs direct immune responses to infections, little is known about their significance in immunization-induced immunity (10–17). Kaufmann et al. demonstrated that Bacillus Camette-Guerin (BCG) vaccination “educates” HSPCs to form components of innate immunity responsible for long-term immune protection from tuberculosis (9). Herein, we demonstrate that vaccine composition influences HSPC frequency by eliciting a unique transcriptional landscape that impacts both innate and adaptive immunity. Recent re-emergence of pertussis, a highly contagious respiratory infection caused by the Gram-negative bacterial pathogen *Bp*, prompted our use of a pertussis model to investigate the roles of HSPCs in vaccine efficacy. Immunization strategies were altered from the use of highly efficacious whole cell vaccines (WCVs), such as DTP, to acellular vaccines (ACV) (DTaP/tdap) in the 1990s. Evidence suggests that recent increases in pertussis cases are due in part to waning ACV protection (18–20). Epidemiological data indicate that when ACVs prevent disease, they may fail to completely prevent infection or transmission (18, 19). Without enhanced vaccines, pertussis incidence is expected to continue to rise, given that a greater proportion of the population over time will only be ACV-immunized. Because WCVs offer longer-lived protection (21), comparisons regarding how immune responses to ACV and WCV differ, including impact on HSPCs, may provide insight for next generation ACVs that are safe and efficacious.

To investigate the role of vaccine content on influencing HSPCs, it is essential to consider known components of each pertussis vaccine. Despite differences in overall antigenic content, WCVs, which are inactivated entire *Bp* organisms, and ACVs, which contain purified components of *Bp*, share some common antigens: pertussis toxin (PT), filamentous hemagglutinin (FHA), pertactin, and fimbriae. ACVs contain aluminum hydroxide, which contributes to a T helper (Th)2-polarized response. Conversely, lipo-oligosaccharide (LOS) in WCVs promotes Th1/Th17 responses (22) despite DTP combined vaccines' inclusion of alum. Little is known regarding the influence of the components of pertussis vaccines on HSPCs. Enzymatically active PT can induce HSC mobilization by blocking G protein function (23), but it is inactivated in ACVs and present only in low concentrations in WCVs (unpublished observations). Additionally, alum, which is present in ACVs and some WCVs, is known to induce granulopoiesis (24) while LOS, present only in WCVs, stimulates TLR4 signaling known to induce HSPC expansion in bone marrow (25).

In the present study, we tested the hypothesis that vaccine composition determines immune response by HSPCs. To do so, we assessed the impact ACVs and WCVs on HSPC expansion, differentiation, and downstream peripheral immune response. We demonstrate that WCVs (1) induce HSPC expansion, priming them for antigen processing and presentation, (2) increase extramedullary hematopoiesis providing stores of immature immune cells released by the spleen upon infection, and (3) prime HSPCs for rapid B cell maturation upon subsequent *Bp* challenge. We also show that ACVs have little impact on HSPCs. This knowledge provides insights into methods by which immune responses to ACVs and WCVs differ, thus introducing a novel area of study that may explain in part why waning immunity occurs with ACVs.

## Methods

### Mouse models

Phosphate buffered saline (PBS), 1/5th human dose of INFANRIX (GSK) human vaccine (DTap/ACV), or 1/5th human dose of National Institute for Biological Standards and WHO International Standard *Bp* vaccine (NIBSC 94-532) (WCV) were used to immunize 5-weeks-old female CD-1 mice (Charles River) by intraperitoneal injection (Table S1). Anesthetized mice were infected by intranasal administration of 2 × 10^7^ colony forming units (CFU) of *Bp* strain UT25 (26) in 20 μl of PBS. *Bp* was handled using standard biosecurity and institutional procedures necessary for the use of a BSL-2 microorganism. These procedures were approved by the West Virginia University Institutional Biosafety Committee (protocol 17-11-01). Post-vaccination and challenge, organs/tissues were extracted in sterile conditions from mice euthanized by pentobarbital injection as recommended by the Panel on Euthanasia of the American Veterinary Medical Association. Cardiac puncture blood was collected. Serum was separated by centrifugation using Microtainer blood collection tubes (BD). Trachea and lungs were homogenized to determine bacterial burden. For *Bp* burden in the nares, 1 ml of PBS was flushed through the nares. Serial dilutions in PBS were plated on Bordet Gengou (BG) Agar containing 15% sheep's blood and streptomycin (100 μg/ml). Bone marrow extraction was performed by flushing RPMI media (ATCC) + 10% fetal bovine serum (FBS) (Sigma) through mouse femurs. Cells were pelleted by centrifugation (500 g) and red blood cell lysis was performed by incubating cells in 1 ml 1X BD PharmLyse™ (BD Biosciences) for 2 min at 37°C. Cells were washed in 1X PBS containing 2% FBS and prepared for flow cytometry. Spleens were dissociated by being passed through a 70 μm mesh filter in 5 ml RPMI media (ATCC) + 10% FBS (Sigma) with the aid of a syringe plunger. Cells were pelleted by centrifugation (500 g) and red blood cell lysis was performed by incubating cells in 1 ml 1X BD PharmLyse™ (BD Biosciences) for 2 min at 37°C. Cells were washed in 1X PBS containing 2% FBS and prepared for flow cytometry. This study was reviewed and approved by the West Virginia University Institutional Animal Care and Use Committee (protocol Damron 14-1211).

### Complete blood counts and histology

Complete peripheral blood (PB) counts were analyzed using a Drew Scientific Hemavet 950. Briefly, a portion of PB from the cardiac puncture was collected using Microtainer blood collection tubes (BD) with a K2EDTA additive. After thorough mixing within the K2EDTA containing tube, blood was transferred to a 1.5 ml microcentrifuge tube and run on the Drew Scientific Hemavet 950. For tissue histology, tibias and spleens were fixed in formalin, embedded in paraffin blocks, sectioned, and stained with hematoxylin and eosin (H&E).

### Flow cytometry

Following Fc receptor blocking in PBS containing 2% FBS, 1 × 10^6^ cells were incubated in antibody for 1 h at 4°C in the dark. Cells were then washed 2 times with PBS, and resuspended in fluorescence-activated cell sorting (FACS) buffer. Flow cytometry antibodies (listed in Table S2) were chosen to acquire a broad understanding of how different vaccine compositions influence the development of both innate and adaptive immune cells. FACS analyses were performed on BD LSRFortessa or FACSAria III cytometers. Data was analyzed using FlowJo_V10.

### Cytokine analysis

Spleen homogenates were pelleted by centrifugation (1,000 g for 5 min). Collected supernatant was stored at −80°C until analysis. Concentrations of cytokineswere determined by quantitative sandwich immunoassays, Meso Scale Discovery (Rockville, MD) V-PLEX Proinflammatory Panel (K15048G-1).

### Isolation of RNA, illumina library preparation, and sequencing

RNA was prepared immediately using RNeasy purification kits (Qiagen) and quantified using Qubit 3.0 (ThermoFisher). RNA integrity was assessed using Agilent BioAnalyzer RNA Pico chip. Samples were submitted for Ribo-zero rRNA depletion (Illumina) and reassessed for RNA integrity. Samples were processed into libraries by the Ovation RNA-Seq System v2 (NuGEN) protocol. Quality control was performed on libraries using the KAPA qPCR QC assay (KAPA Biosystems). Libraries (36 total) were sequenced on an illumina HiSeq 1500 at the Marshall University Genomics Core facility, 2 × 50 bp resulting in ~16 M reads per sample. Sequencing data was deposited to the Sequence Read Archive (reference number SRP130256, BioProject number PRJNA430726).

### RNAseq bioinformatic analyses

Reads were analyzed using CLC Genomics Workbench 9.5. Default settings were used for mapping reads against the GRCm38 *mus musculus* genome. Fold changes in gene expression and statistical analyses were performed using Extraction of Differential Gene Expression (EDGE) test on *p*-values. Venn diagrams and gene set enrichment were established using Venny 2.1 (27) and PANTHER, respectively. Significant data was determined by FDR (<0.05). In Ingenuity Pathway Analysis (IPA), significant genes (*p* < 0.05) were uploaded into software and datasets were compared across immunization and challenge conditions. IPA's Upstream Regulator analytic was utilized to identify upstream transcriptional regulators.

### Immunoglobulin and B- and T-cell receptor profiling

B cell clones were identified using MiXCR software (MiLabratory) (28), capable of generating quantitated clonotypes of immunoglobulins. Reads were merged using concatenation and imported into MiXCR software and aligned to each other to generate clonotypes based on Variable (V), Diversity (D), and Joining (J) (VDJ) segment regions of unique immunoglobulins specific to each sample. Clone data was grouped based on vaccine received and time point. Clonotypes were separated into T and B cells based on T-cell receptor or B-cell receptor specific sequences. Prepared data files were imported into VDJtools (MiLabratory) for data representation as previously described (29).

### Statistical analysis

Results are depicted as mean ± SEM. Excluding RNAseq statistics described above, all other statistical analyses were performed using two way ANOVAs and Tukey's multiple comparisons. GraphPad Prism software was used for statistical analysis.

## Results

### Vaccine content determines peripheral immune response to *Bp* challenge

**Figure 1:**
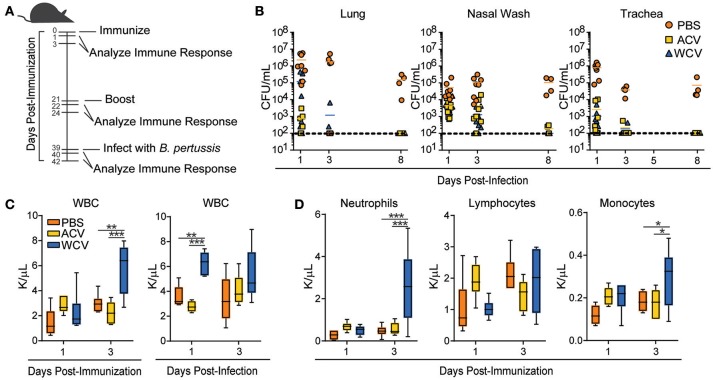
Vaccine content determines PB immune response to *Bp* challenge. **(A)** The workflow for immunization and infection schedules of 5-weeks-old female CD-1 mice is represented. Mice were immunized with phosphate buffered saline (PBS), *Bp* acellular vaccine (ACV), or *Bp* whole cell vaccine (WCV) and infected with 2 × 10^7^ CFU UT25 *Bp*. Immune responses in mice were evaluated at 1 and 3 days after immunization, boost, and infection. **(B)** *Bp* bacterial burden was measured in lung, nasal wash, and trachea of mice (*n* = 4–8/group for each time point) by counting CFUs produced 3 days after plating and incubating serial dilutions of homogenates on BG containing streptomycin (100 μg/ml) at 37°C. **(C)** White blood cell counts were measured (*n* = 5–6/group for each time point) on a Drew Scientific Hemavet 950 at days 1 and 3 post-immunization and days 1 and 3 post-infection **p < 0.01; ***p < 0.001. **(D)** Differential white blood cell counts measured on days 1 and 3 post-immunization are represented. *p < 0.05; 2-way ANOVAs with Tukey's multiple comparisons. Bars extend to represent minimum to maximum values, while the line represents the mean.

To investigate how vaccine impact on HSPCs contributes to immunity to pertussis in CD-1 outbred mice, we utilized a vaccination and challenge workflow (Figure 1A) for profiling immune responses after primary and boost immunizations as well *Bp* challenge. Five-weeks-old mice were immunized with PBS (vehicle control), ACV, or WCV (Table S1), boosted, and subsequently challenged with *Bp*. Although ACVs wane in immune protection over time in humans (18–20), short-term experiments in mice show similar initial clearance of bacterial burden in sites of respiratory infection (lung, nasal wash, and trachea) in both groups (Figure 1B) with 1/5th human doses. Serological analysis of Ig(A+G+M) indicates that only the ACV induces detectable anti-PT, whereas both the ACV and WCV induce anti-FHA. As expected, the LOS-containing WCV induces production of anti-LOS (serology data not shown).

To demonstrate consistency of our model with other groups, we assessed PB cell counts. Pertussis causes leukocytosis in humans, rodents, and baboons (30–32), and WCV immunization has been shown to also result in leukocytosis (33). Our WCV-immunized mice exhibit increases in white blood cells at day 3 post-immunization when compared to PBS and ACV-immunized mice. WBCs increase again following subsequent infection (day 1) (Figure 1C) in WCV-immunized mice. Increases in WBCs are attributed to increased myeloid cells, particularly neutrophils and monocytes (Figure 1D). Mimicking *Bp* infection, our WCV immunization model induces leukocytosis in response to immunization. These data directed our attention to upstream events leading to WBC responses.

### WCV content alters the size and composition of peripheral lymphoid organs

**Figure 2:**
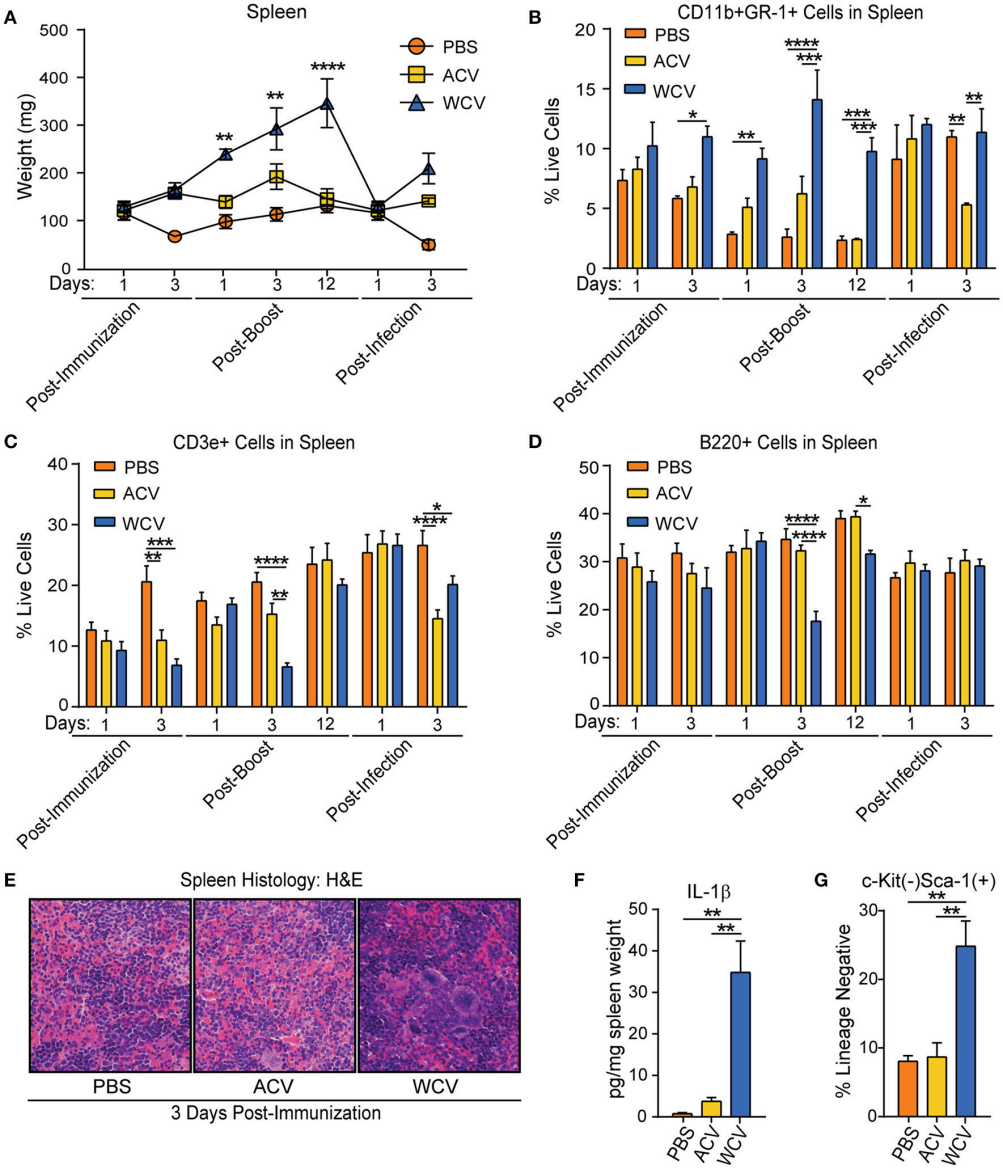
WCV content induces alterations in the size and composition of peripheral lymphoid organs suggesting extramedullary hematopoiesis may occur. **(A)** Spleen weights from CD-1 mice (*n* = 4/group for each time point) immunized with phosphate buffered saline (PBS), *Bp* acellular vaccine (ACV), or *Bp* whole cell vaccine (WCV) were recorded at the indicated time points across the immunization and infection schedule. **(B)** CD11b^+^Gr-1^+^ cell proportions in the spleen (*n* = 4/group for each time point) were measured by flow cytometric analysis. **(C)** CD3e^+^ cell proportions in the spleen (*n* = 4/group for each time point) were measured by flow cytometric analysis. **(D)** B220^+^ cell proportions in the spleen (*n* = 4/group for each time point) were measured by flow cytometric analysis. **(E)** Representative images were taken of H&E-stained spleen (*n* = 4/group) at 400X magnification. **(F)** IL-1β in spleen homogenate (*n* = 4/group) was measured by quantitative sandwich immunoassays. G. Proportions of lineage^−^c-kit^−^Sca-1^+^ cells in the spleen (*n* = 4/group for each time point) were measured using flow cytometric analysis. *p < 0.05; **p < 0.01; ***p < 0.001; ****p < 0.0001; 2-way ANOVAs with Tukey's multiple comparisons. Error bars are mean ± SEM values.

To determine how each vaccination further effects the periphery, evaluations of lymphoid organs were performed. Two major lymphoid organs, which defend the body against invading pathogens include the thymus and the spleen. Thymus and spleen weights were recorded throughout our experimental schedule. Though only minor differences occur between vaccination groups in thymus weight and cell population proportions (data not shown), spleen sizes (Figure S1A), and weights (Figure 2A) increase progressively post-immunization with the WCV when compared to other groups. Cell counts following RBC lysis confirm increased spleen cellularity in WCV-immunized mice (data not shown). Spleens then decrease dramatically and rapidly in size and weight (2.45-fold) upon *Bp* infection (Figure S1A and Figure 2A), suggesting that cells migrate from the spleen to sites of infection. Similarly, naïve infected mice also exhibit decreases in spleen weight (1.94-fold) upon infection, but this occurrence is delayed when compared to WCV-immunized mice. Naïve mice exhibit a reduction in spleen weight 3 days post-infection, whereas WCV-immunized mice exhibit reduced spleen weight at 1 day post-infection. By day 3 post-infection spleen sizes are beginning to increase again in WCV-immunized mice. Flow cytometric analysis of splenocytes revealed that CD11b^+^GR-1^+^ myeloid cell proportions (Figure S1B and Figure 2B) are significantly increased in WCV-immunized mice, suggesting that they contribute to increased spleen size. CD11b^+^GR-1^+^ cells comprise a heterogeneous population of myeloid cells including myeloid progenitors, immature macrophages, immature granulocytes, and immature dendritic cells (34).

WCV-immunized mice display proportional decreases in splenic CD3e^+^ T cells (post-immunization day 3, post-boost day 3, and post-infection day 3 and B220^+^ B cells (post-boost days 3 and 12) (Figures 2C,D). To determine if proportional decreases were due only to reflect the increased myeloid cells in the spleen, total B and T cells were assessed in the spleen at days 1 and 3 post-immunization and post-infection (Figures S1C,D). Total B cells do not differ between groups post-immunization. T cell proportional decreases in WCV-immunized mice, however, are due in part to a decrease in total T cells at day 3 post-immunization. Upon subsequent infection (day 3), however, CD3e^+^ T cells are present in higher proportions and total numbers in the spleen in PBS and WCV-immunized mice when compared to ACV-immunized mice (Figure S1C and Figure 2C). Interestingly, as myeloid cell proportions become similar to PBS and ACV-immunized mice following infection, both T and B cells are increased in total number in the spleen. To further elucidate to impact of immunization formulation on cell population dynamics and cellularity in the spleen, H&E staining of spleen cross sections (day 3 post-vaccination) were performed. This examination of histology confirms hypercellularity and increases in myeloid cells, including megakaryocytes, occurs in WCV-immunized mice when compared to other groups (Figure 2E).

Parallel to increases in myeloid cell proportions in the spleens of WCV-immunized mice, on post-immunization day 3, we observed a significant increase in splenic IL-1β production (Figure 2F). IL-1β, which is produced by myeloid cells, enhances lymphocyte processes such as antigen-primed CD4 and CD8 T-cell expansion, differentiation, and migration to the periphery as well as memory (35, 36). Additionally, on post-immunization day 3, when compared to PBS and ACV groups, WCV-immunized mice exhibit larger proportions of splenic Lineage^−^Sca-1^+^c-Kit^−^ cells (Figure 2G), which have been described as lymphoid progenitor cells that expand in the spleen upon infection, preferentially maturing into B cells (37, 38). Taken together, these data suggest that extramedullary hematopoiesis, which can occur due to mobilization of bone marrow HSPCs to the spleen (39, 40), is expanded in WCV immunizations. Evidence gathered from kinetics of immature cell population changes in the spleen suggest that bone marrow HSPC compartment composition may play a role in splenic immune response to vaccination.

### WCV immunization induces MPP expansion in the bone marrow

**Figure 3:**
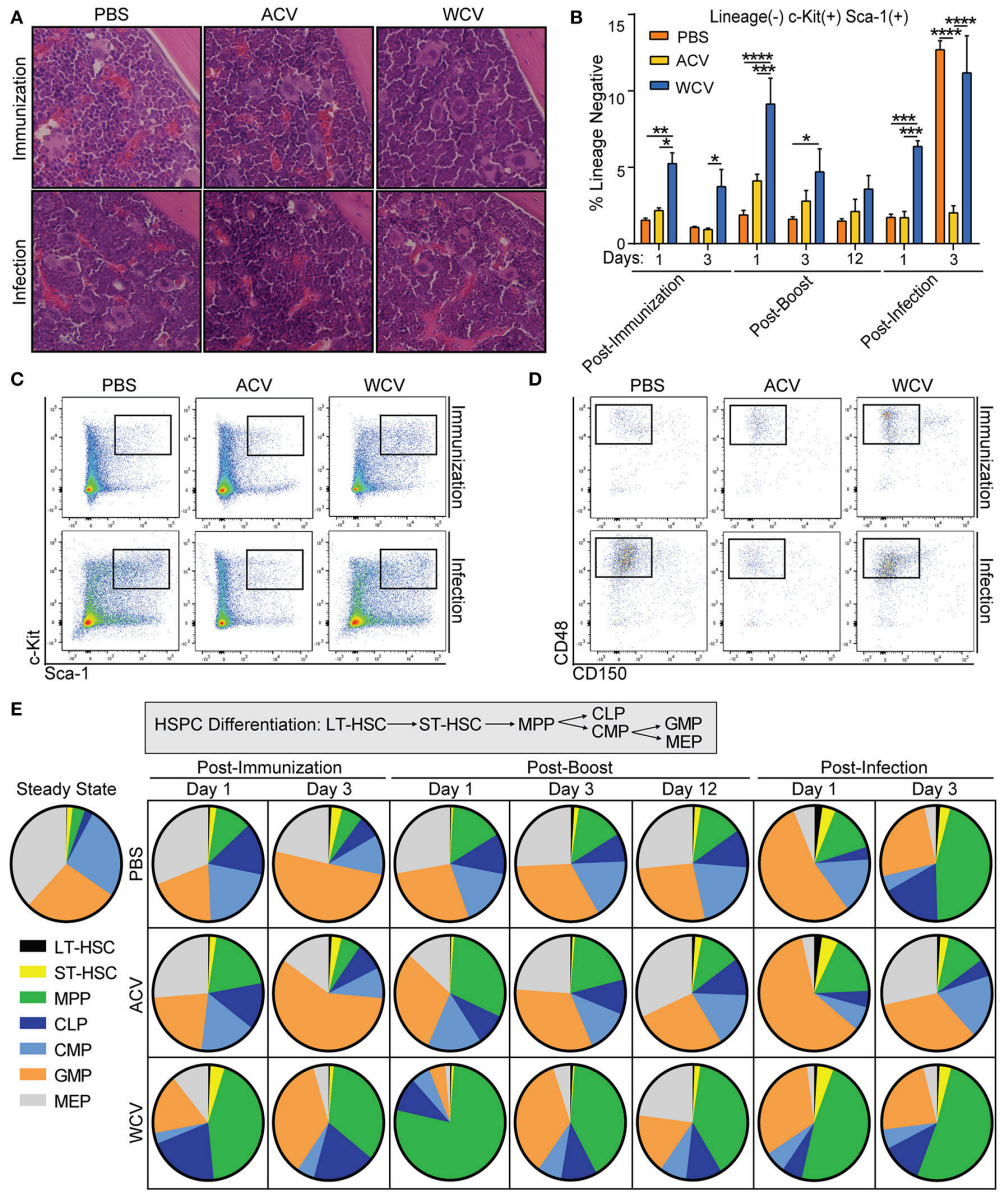
WCV immunization induces MPP expansion in the bone marrow. **(A)** Representative images of cross sections of tibia from CD-1 mice (*n* = 4/group for each time point) immunized with phosphate buffered saline (PBS), *Bp* acellular vaccine (ACV), or *Bp* whole cell vaccine (WCV) and infected with 10^7^ CFU *Bp* were taken at 400X magnification. **(B)** Proportions of lineage^−^Sca-1^+^ckit^+^ (LSK) cells were evaluated in the bone marrow (*n* = 4/group for each time point) using flow cytometric analysis. **(C)** Representative dot plots of the flow cytometric analysis from 3B are shown. **(D)** Representative dot plots from further gating on LSK proportions to show CD48 and CD150 biexponential plot. **(E)** Hematopoietic stem and progenitor cell proportions were analyzed by flow cytometry and are represented as parts of the whole. *p < 0.05; **p < 0.01; ***p < 0.001; ****p < 0.0001; 2-way ANOVAs with Tukey's multiple comparisons. Error bars are mean ± SEM values.

All groups evaluated have a similar level of cellularity in the bone marrow following infection as evidenced by H&E staining of lengthwise cross-sections of tibia (Figure 3A) and total cell counts (data not shown). Flow cytometric analysis of bone marrow HSPCs demonstrates that Lineage-Sca-1^+^c-Kit^+^ (LSK) cell proportions increase in WCV-immunized mice when compared to PBS and ACV-immunized mice (Figures 3B,C). Interestingly, naïve mice elicit robust delayed LSK expansion upon *Bp* challenge (Figure 3B). Further analysis of LSK cells in WCV and naïve challenge indicate that the expanded LSK are CD48^+^CD150^−^ multipotent progenitor cells (MPPs) (Figure 3D). To gain insight into how proportions of these progenitors change among groups when compared to proportions of other populations, LT-HSC, ST-HSC, MPP, common lymphoid progenitor (CLP), common myeloid progenitor (CMP), granulocyte monocyte progenitor (GMP), and megakaryocyte erythrocyte progenitor (MEP) bone marrow populations were analyzed to be presented as parts of the whole HSPC compartment (Figure 3E). Major differences in HSPC proportions occur across vaccine groups. WCV-immunized mice exhibit large proportions of MPPs upon initial vaccination (day 1) and boost (day 1) that become reduced with time after each incidence (day 3 post immunization and boost), while ACV-immunized mice exhibit MPP proportions only slightly larger than those of PBS control (day 1 post-immunization, day 1 post-boost). It is evident that WCV-immunized mice post-vaccination (day 1) highly resemble naïve infection (PBS) post-challenge (day 3).

### WCV immunization primes bone marrow HSPCs for rapid maturation of developing B cells upon subsequent infection

**Figure 4:**
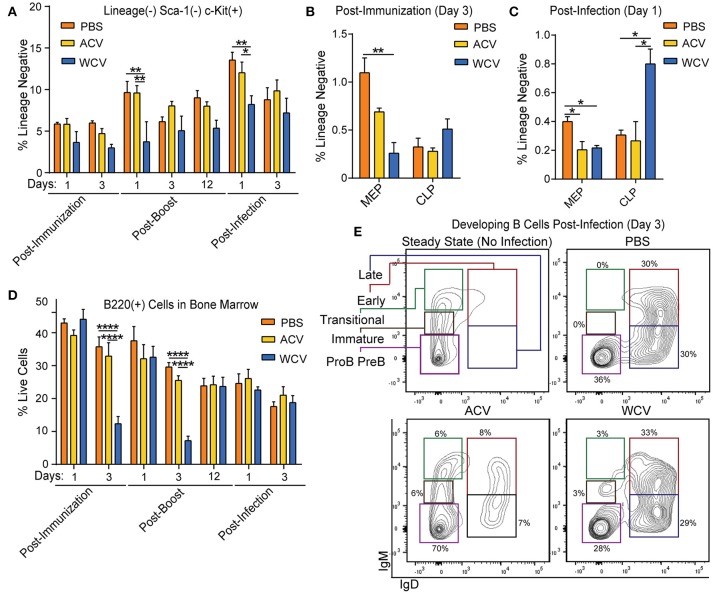
WCV immunization prepares HSPCs for rapid maturation of developing B cells upon subsequent infection. **(A)** Proportions of lineage^−^Sca-1^+^ckit^+^ (LK) cells were evaluated in the bone marrow of CD-1 mice (*n* = 4/group for each time point) immunized with phosphate buffered saline (PBS), *Bp* acellular vaccine (ACV), or *Bp* whole cell vaccine (WCV) and infected with 10^7^ CFU *Bp* using flow cytometric analysis. **(B)** Proportions of megakaryocyte erythroid progenitors (MEPs) and common lymphoid progenitors (CLPs) were evaluated in mouse bone marrow (*n* = 4/group for each time point) post-immunization and **(C)** post-infection by flow cytometric analysis. **(D)** Proportions B220^+^ cells were evaluated in mouse bone marrow (*n* = 4/group for each time point) using flow cytometric analysis. **(E)** Representative dot plots from further gating on cells from part D are shown. *p < 0.05; **p < 0.01; ****p < 0.0001; 2-way ANOVAs with Tukey's multiple comparisons. Error bars are mean ± SEM values.

In WCV-immunized mice, Lineage^−^Sca-1^−^c-kit^+^ (LK) cell proportions are decreased upon boost and infection when compared to other groups (Figure 4A). Further analysis into the cell populations that comprise LK cells revealed a proportional decrease within WCV-immunized mice in MEP populations post-immunization (day 3) and post-infection (day 1) (Figures 4C,D). Regarding lymphoid progenitors, an increase in CLP proportions upon infection is observed in WCV-immunized mice (Figure 4C). Aside from B cells, no mature populations evaluated differed across groups (data not shown). B220^+^ cell proportions, however, were reduced significantly in WCV mice post-immunization (day 3) and post-boost (day 3) (Figure 4C). Interestingly, post-infection (day 3), B220^+^ cells in WCV-immunized mice showed signs of robust maturation processes (Figure 4E). Our data indicate that HSPC expansion in response to immunization and infection is a dynamic process, which prompted us to investigate the transcriptomic profiles of HSPCs to better understand the mechanisms and systems driving observed responses.

### RNAseq analysis of developing VDJ recombination in HSPCs

**Figure 5:**
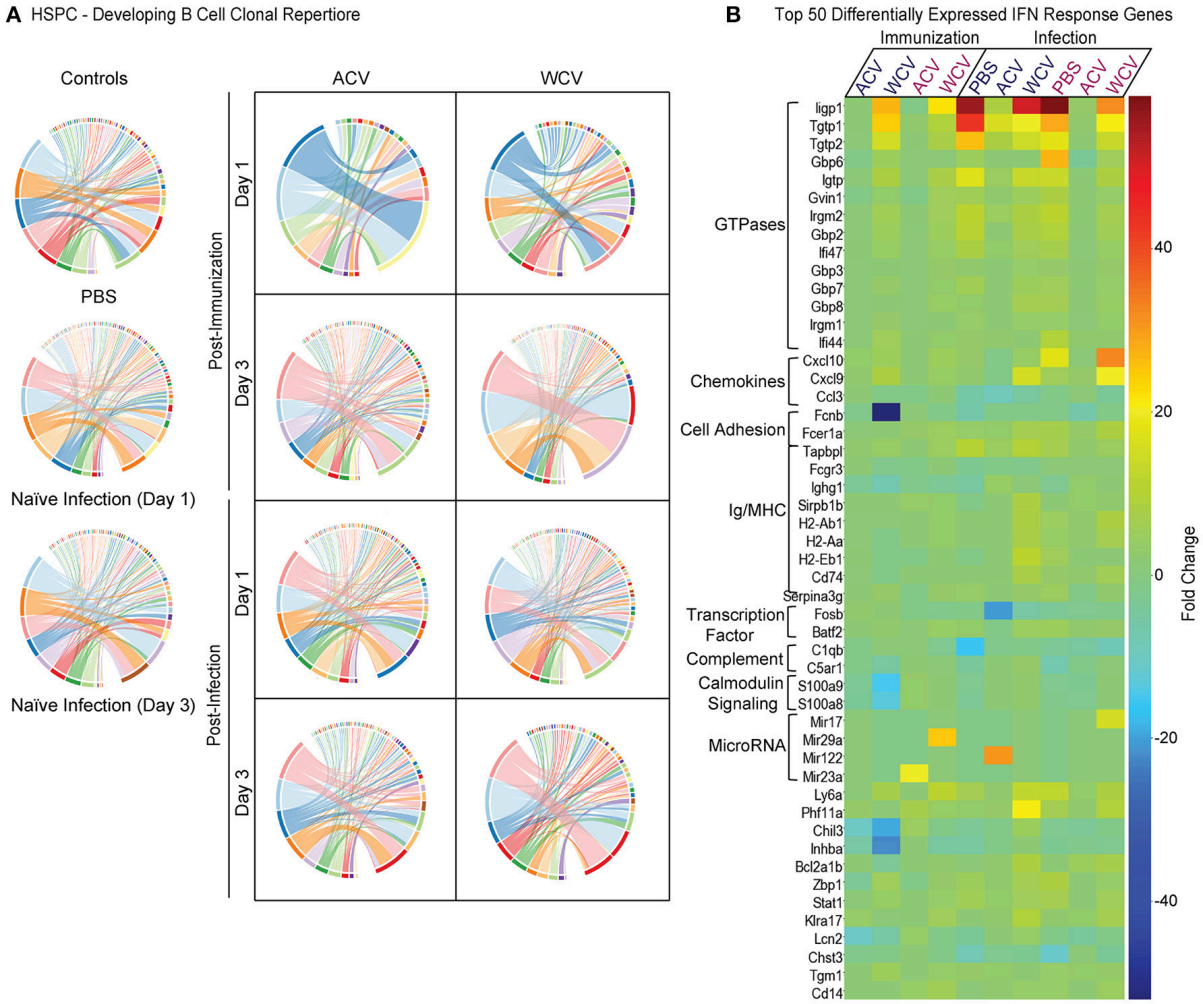
Vaccine content drives the transcriptional landscape of HSPCs. **(A)** Pairwise overlap circos plots of HSPC B cell clonal repertoires (*n* = 6mice/group) were prepared using MiXCR software. Count, frequency and diversity panels correspond to the read count, frequency (both non-symmetric) and the total number of clonotypes that are shared between samples. Pairwise overlaps are stacked, i.e., segment arc length is not equal to sample size. **(B)** A heatmap was prepared to represent the top 50 differentially expressed genes downstream of IFNα, β, and/or γ across all immunization and infection groups. Day 1 for immunization and infection is shown below the dark blue text, while day 3 is shown below the magenta text. Gene sets for the heatmap were prepared using IPA's Upstream Regulator analytic tool. RNA was pooled from 2 mice for each sample n.

To characterize gene expression profiles of HPSCs in relation to immunization and challenge, HSPCs were isolated from the bone marrow of mice upon immunization and infection. Isolated RNA was converted into libraries for Illumina platform sequencing. We obtained expression data for ~60% of all encoded murine genes across all experimental groups (Table S3) indicating robust transcriptomic analysis. Since differential expansion of bone marrow B cell populations occurs in response to immunization (Figure 4), we hypothesized that B cell population dynamics coincide with differential VDJ recombination developing within HSPCs. VDJ recombination and somatic hypermutation during B cell development and affinity maturation are responsible for diversity of antigen-recognizing B cell receptors (BCRs). Highly variable complementarity determining region 3, which plays a critical role in antigen specificity and binding affinity, is located at the VDJ junction BCR heavy chains (41). Mapped reads were analyzed with MiTCRx and VJRtools to identify immunoglobulin sequences, building libraries of clonal types. Figure 5A shows resulting B cell repertoires for all immunization and infections conditions. Spectral plots show that both ACV and WCV immunization influence clonal populations compared to naïve (PBS) mice. Post-immunization (day 3), these clone repertoires return to a state more similar to naïve mice. Upon infection, the clone repertoires diversify and it becomes difficult to distinguish naïve infected or immunized groups. Investigation of per clone diversity revealed that only the WCV group exhibits enhanced amounts of any one clone. The IGKV15-103/IGKJ4 clone was enriched in HSPCs from WCV-immunized mice and WCV-immunized mice that were subsequently infected. We speculate that this clone may be responsible for anti-LOS which is produced in high quantities in WCV-immunized mice. Enlarged spectral plots with labeled clones can be found in Figures S2–S4.

### Immunization and infection induces unique gene expression profiles of HSPCs

RNA sequencing (days 1 and 3 post-immunization, and days 1 and 3 post-infection) demonstrates that HSPCs respond to WCV-immunization by altering many immune response gene signatures. WCV-immunized mice exhibit >19-fold more differentially expressed genes when compared to ACV-immunized mice (Figure S5A). The most enriched of 18 significant gene signatures (fold change > 5) in this WCV HSPC gene set include protein folding, antigen processing and presentation, and response to interferons (IFNs) (Figure S5B). Genes differentially expressed only in HSPCs of ACV-immunized mice as well as genes differentially expressed in both immunization groups yielded no significantly enriched gene signatures.

Differential gene expression between vaccine groups upon challenge with *Bp* reveals that 35 genes overlap all groups, 88 genes exhibit unique overlap of naïve infection (PBS-immunized) and WCV, while 9 genes overlap ACV and naïve infected mice (Figure S5C). Enrichment of 123 genes differentially expressed in HSPCs from naïve infected mice that do not overlap with other groups resulted in signatures involving phagocytosis, complement, regulation of B cell activation, defense response to bacterium, and innate immune response (Figure S5D). Enrichment of genes uniquely overlapping naïve infection and WCV-immunized, *Bp*-infected mice produce similar defense response signatures (Figure S5E), but additionally include MHC class I and II processing and presentation and response to IFNs. No enriched pathways exist for differentially expressed genes that overlap naïve infection and ACV-immunized, *Bp*-infected mice. Additionally no enriched pathways exist for gene sets unique to WCV or ACV. Analysis of 35 genes overlapping all infection groups presents 8 significant gene signatures that resemble those observed in WCV immunization only (Figure S5F). Direct comparisons of initial immunization to naïve infection reveal that gene signature responses in HSPCs upon naïve infection resemble those of WCV-immunized, non-challenged mice as well. These include IFN response, MHC class I and II molecule production, chaperone folding, and immunoproteasome formation (data not shown), suggesting increased antigen processing and presentation.

Further highlighting mechanisms driving these immune responses, powerful algorithms and advanced analysis capabilities available through IPA allowed for the identification of transcriptional patterns that indicate a significant role for IFN stimulated gene (ISG) expression in HSPCs from WCV-immunized mice when compared to ACV-immunized mice. Z-scores, used to infer the activation state of predicted upstream transcriptional regulators, were increased in HSPCs from WCV-immunized mice for upstream IFNα, IFNβ, and IFNγ signaling when compared to ACV-immunized HSPCs. Similar to immunization, naïve infection and challenged WCV-immunized mice exhibited increased activation of these pathways compared to ACV-immunized mice. A heatmap displaying the top 50 differentially expressed IFN (α, β, and γ) stimulated genes is shown in Figure 5B.

**Figure 6:**
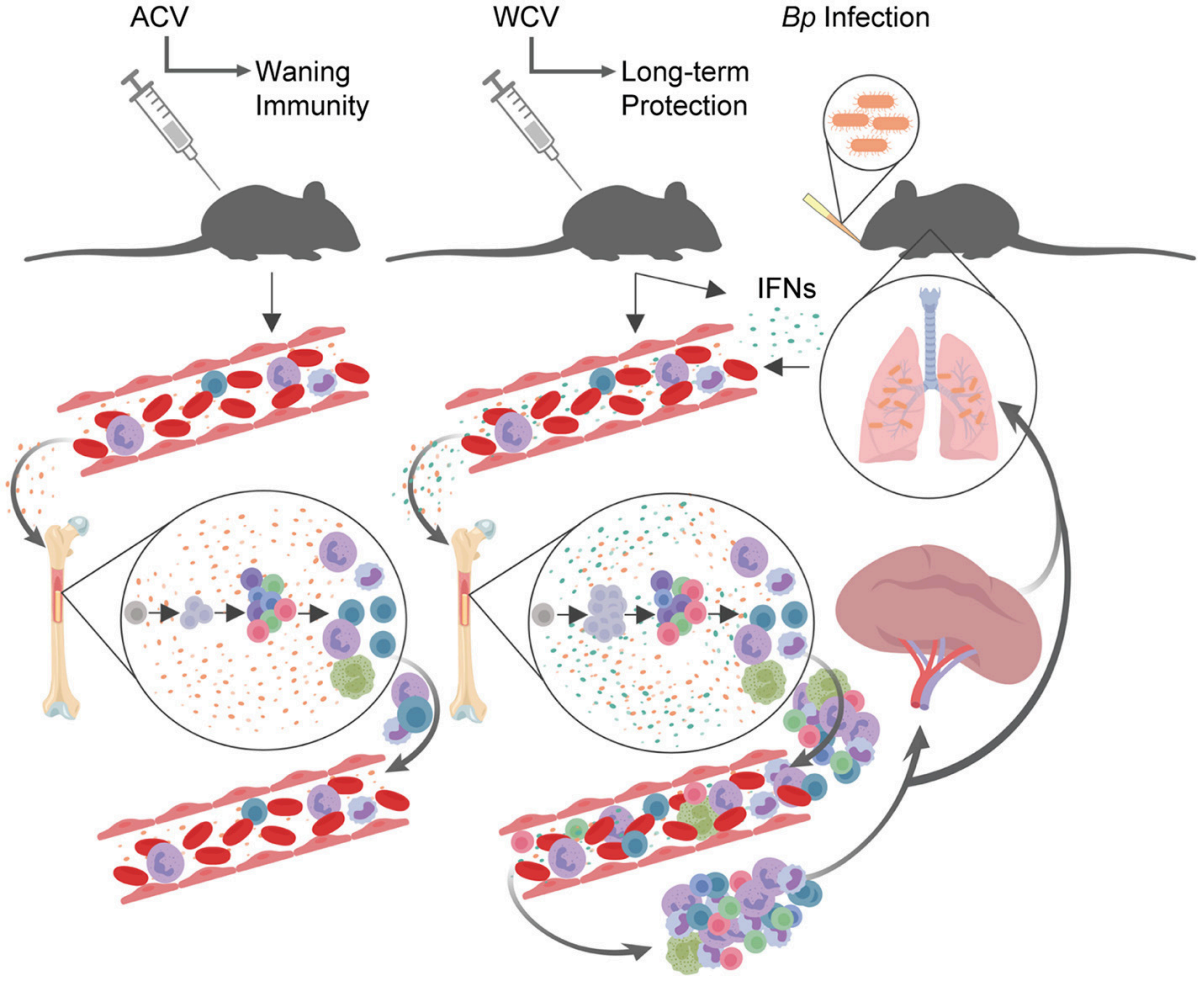
Proposed model for vaccination and infection effects on HSPCs and downstream immune response. Following vaccination with ACV, similar to PBS (negative control) immunized mice, cytokines in the peripheral blood impact the bone marrow microenvironment to produce immune cells. Following vaccination with WCV, IFNs are produced and circulate in the blood, impacting the bone marrow microenvironment, thereby directing an expansion of HSPCs. In addition to HPSC proportional expansion, mature B cells decrease proportionally within in the bone marrow, while all other immune cells analyzed (T cells, macrophages, and granulocytes) do not significantly change. Given that bone marrow cellularity remains similar to PBS and ACV immunization groups, we hypothesize that upon production, select progenitors and differentiated cells move out of the marrow and into the peripheral blood where they migrate to the spleen. Upon subsequent infection, HSPCs undergo expansion again and cells in the peripheral blood and spleen migrate to sites of infection to clear *Bp*. During naïve infection, a similar process occurs. This model was created using BioRender.

Our proposed model for vaccine content influence on HSPCs through IFN signaling pathways is described in Figure 6. Briefly, we have propose that following vaccination with ACV, similar to PBS—immunized mice, cytokines in the PB impact the bone marrow microenvironment to produce immune cells required for homeostasis. Following vaccination with WCV, however, we speculate that additional IFNs are produced and circulate in the blood, impacting the bone marrow microenvironment, thereby directing an expansion of HSPCs. In addition to HPSC proportional expansion, we observe that mature B cells decrease proportionally within in the bone marrow, while all other immune cells analyzed (T cells, macrophages, and granulocytes) do not significantly change. Given that bone marrow cellularity remains similar to PBS and ACV immunization groups, we hypothesize that upon production, select progenitors and differentiated cells move out of the marrow and into the PB where a subset of these cells migrate to the spleen. Upon subsequent infection, HSPCs undergo expansion again and cells in the PB and spleen migrate to sites of infection to clear *Bp*. During naïve infection, a similar process occurs. We propose that immune cell activation in the periphery results in IFN signaling that reaches HSPCs of the bone marrow, inducing ISG expression and influencing cell cycle, survival, autophagy, and innate immune signaling pathways among others.

## Discussion

Investigating the impact of altered HSPC populations following immunization is imperative to advancing knowledge that will inform the design of future vaccines. We have described how content of pertussis vaccines influences HSPC population kinetics and associated peripheral immune responses. While WCVs or ACVs allow mice to clear short-term infection, longevity of immune protection may reside in how HSPCs are influenced following immunization. This includes the propensity of these cells to undergo expansion and priming for future infection responses. Our data indicate that LSK cell populations are increased in the bone marrow following WCV immunization. This phenomenon mimics how LSK cells are impacted in response to naïve infection. HSPCs have the capacity to differentiate into all types of immune cells, and their expansion following immunization with an efficacious vaccine or naïve infection correlates with immune cell population changes in the PB and organs. Further investigation of the HSPC compartment revealed that LSK expansion is largely due to an increase in MPPs. Since MPPs support the generation of all types of mature blood cells, it is not surprising to find that cell populations of both myeloid and lymphoid lineages are altered in the bone marrow, blood, or spleen.

Lymphoid progenitor cell population increases in the bone marrow and spleen following WCV immunization suggest that greater proportions of B and T cells are undergoing early stages of development following WCV immunization when compared to PBS and ACV immunization. Furthermore, cytokines produced by the multitude of myeloid cells in the blood and spleen could impact the way in which these developing lymphocytes differentiate. Future studies will include kinetics of B and T cell development for several weeks following immunization. Regarding immature myeloid cell population increases in the spleen following immunization, these cells most likely produced by extramedullary hematopoiesis, seem to act as a reservoir, poised to move out to the respiratory tract upon subsequent infection.

In revealing molecular mechanisms responsible for HSPC alterations and downstream immune cell population changes observed, transcriptional signatures from HSPCs isolated from WCV immunization and naïve infection fall into interesting immune-related gene families. Involved in autophagy, immunoproteasome formation, molecular chaperone processes, and class I and class II MHC molecule components, these gene families suggest that HSPCs may be primed for antigen presentation. Taken together with the dynamics of B cell development and developing VDJ recombination within HSPCs, these data support a role for HSPC involvement in influencing long-term immune protection via impact on MHC class II antigen presenting B cell development.

The most striking gene expression change observed in infection and WCV immunization, but not acellular vaccination, involves IFN-inducible genes (Figure 5B). Type 1 and 2 IFNs are known for inducing HSC proliferation, which potentially explains bone marrow HSPC expansion (42–44). Essers et al. showed that in response to IFNα treatment, HSPCs exhibit increased Sca-1 and phosphorylation of STAT1 and PKB/Akt. Furthermore, they and others established that STAT1 and Sca-1 mediate IFNα-induced HSC proliferation. We speculate that signaling cascades downstream of IFNs, such as mTOR, PI3K, MAPK, and NFκB pathways, which are all affected in our datasets, influence HSPC frequency observed in our models. Beyond impacting cell survival, proliferation, and cell cycle, many IFN-regulated genes implicated in our data are involved in immune response. The immunity-related guanosine triphosphatase (IRG) family, for example, has well-documented roles in clearing infection (45, 46). Among other mechanisms, IRGs interfere with infectious pathogens by disrupting phagocytic vacuoles during infection (45, 46).

Of the IRG family, 47-kDa GTPases in mice include: Irgm1, Irgm2, Ifi47, Tgtp1, Tgtp2, Igtp1, and Igtp2, all of which are upregulated in naïve *Bp* infection and WCV immunization against *Bp*. Genetic deletion of 47-kDa GTPases severely impairs defense against many primarily intracellular pathogens: *Toxoplasma gondii, Trypanosoma cruzi, Leishmania major, Listeria monocytogenes, Mycobacterium* spp., including *Mycobacterium tuberculosis and Mycobacterium avium, Salmonella typhimurium*, and murine cytomegalovirus (46). Host immune clearance of intracellular pathogens is relies upon IFN-mediated cellular immunity(47). Though *Bp* is primarily considered an extracellular pathogen, it has also been shown to survive inside respiratory epithelial cells, human polymorphonuclear leukocytes, and human monocytes/macrophages (48–50). Moreover, Connelly et al. provided evidence that INFγ-induced GTPases play a role in PT-associated responses in the lungs during *Bp* infection (51). Though IFNγ is responsible for many immune responses (47), it has recently gained much attention in how it mediates GTPases to eliminate pathogens that survive inside of a cell. Mitchell et al. has reviewed the processes of immune clearance of intracellular microorganisms that particularly involve IFNγ GTPases (52). The two systems discussed (1) xenophagy and (2) interferon-regulated GTPase promotion of the rupture of pathogen-containing vacuoles and microbial degradation are thought to participate in cross-talk that balances the killing of pathogens within cells and inflammatory responses. Beyond this, methods by which GTPases are involved in protection against pathogens include: (1) trafficking from the endoplasmic reticulum and Golgi to phagosomes, (2) regulating survival of infected host cells, and (3) regulating pools of effector lymphocytes (45, 46). It has been proposed that when coupled with signals from Toll-like receptors, such as those recognizing LOS, the p47 GTPase family provides an enhanced sensory system for recognizing infectious agents as well (53).

Regarding HSPCs, genetic deletion of *Irgm1*, for which *IRGM* is a human ortholog, results in pancytopenia due to inadequate cell expansion when challenged with mycobacterium or *T. cruzi* (54, 55). Given that we observe high expression of IRG genes during HSPC expansion and robust enrichment of IFN- and STAT1-related genes in HSPCs of both naïve infection and WCV immunization, it can be argued that IFN influence on primitive hematopoietic cells is important in expanding bone marrow HSPCs for the promotion of leukocytosis. Feng et al. described an Irgm1 feedback mechanism in Th1 response that promotes antimicrobial function while limiting detrimental effects of IFNγ on effector T lymphocyte survival (56). Perhaps similarly, this and other GTPases upregulated in HSPCs following infection and WCV immunization are involved in peripheral expansion of immune effector cell populations during downstream Th1 responses to intracellular pathogens. Experts in the pertussis field often contribute WCV efficacy to preferential production of Th1 responses to *Bp* (57–60). We speculate that this is partly maintained by upregulated IRGs within HSPCs.

Relevant to our data, IRGs are also implicated in B cell maturation. IFNγ induces pre-B cells to exhibit surface immunoglobulin (61). Simultaneously, IFN-inducible GTPases increase in expression (61). We postulate that IFN signaling during efficacious vaccination mimics naïve infection, leading to various processes that impact HSPCs, including increased IFN-induced GTPase expression, increased priming of cells for antigen presentation, and skewed MPP and B cell maturation patterns. Similar to our studies, Kaufmann et al. found IFN signaling to be essential to LSK expansion following BCG vaccination. In their studies, it was needed to educate macrophages to provide long-term innate immune protection. Our work, together with additional studies in trained immunity (7–9), demonstrates that understanding the mechanisms by which IFN signaling may prime HSPCs to react to specific pathogens once cells have matured is underappreciated, offering a novel area of investigation regarding long-term immunity.

We expect this work highlighting the importance of vaccine content in influencing HSPC characteristics to provide new insights for the development of future pertussis vaccines as well as provide broad applicability to vaccine efficacy. Acellular pertussis vaccines protect mice against challenge in short term immunization/boost/challenge studies. Similarly, in humans, DTaP performed well in short-term clinical trials in Europe (62). However, with time it is clearly appreciated that DTaP/Tdap immunity wanes. We appreciate that there are substantial differences between murine models and human hosts. In the present study, however, we aimed to characterize HSPC expansion on the transcriptomic level. By identifying biomarkers that can be measured as a function of HSPC expansion, we may be able to develop protocols to better monitor early events in immunization for the purpose of developing vaccine formulations that induce longer term memory.

We hypothesize that the use of adjuvants that induce IFN responses and/or TLR4 signaling will enhance ACVs by influencing HSPC priming and expansion. Our future work with the use of rationally designed, functionally diverse lipid A adjuvants (63) will provide answers as to how induction of HSPC expansion in ACV-immunized mice may contribute to downstream immune responses. Once we have elucidated how these adjuvants may enhance vaccine-induced immunity in mice and other animal models, we expect that this knowledge will lead to improved ACV vaccines for use in humans. Furthermore, in future studies we expect to determine how pertussis immunization and subsequent infection impacts overall HSPC health and longevity.

## Author contributions

MV, DB, KD, TW, ES, SB, CE, ME, WW, MB, and FD performed experiments. MV, EN, and FD analyzed results and composed figures. MV and FD designed experiments and composed the manuscript. All authors participated in the review of the manuscript.

### Conflict of interest statement

The authors declare that the research was conducted in the absence of any commercial or financial relationships that could be construed as a potential conflict of interest.

## Supplementary material

Figure S1 WCV induces alterations in spleen size and B and T cell population composition. **(A)**. Spleen sizes from CD-1 mice (n = 4/group for each time point) immunized with phosphate buffered saline (PBS), Bp acellular vaccine (ACV), or Bp whole cell vaccine (WCV) are shown in representative images for the indicated time points across the immunization and infection schedule. **(B)**. Representative dot plots from flow cytometric analysis described in Figure 2B. **(C)**. Total CD3e+ cells in the spleen (n = 4/group for each time point) were calculated by multiplying the percentage of CD3e+ live cells (Figure 2C) in the spleen by total number of live cells in the spleen. **(D)** Total B220+ cells in the spleen (n = 4/group for each time point) were calculated by multiplying the percentage of B220+ live cells in the spleen (Figure 2D) by total number of live cells in the spleen. ^*^*p* < 0.05; ^**^*p* < 0.01; ^***^*p* < 0.001; ^****^*p* < 0.0001; 2-way ANOVAs with Tukey's multiple comparisons. Error bars are mean ± SEM values.

Figure S2 Enlargement of controls for the transcriptional landscape of HSPCs B cell clonal repertoires. Pairwise overlap circos plots of HSPC B cell clonal repertoires (n = 6mice/group) prepared using MiXCR software and shown in Figure 5A were enlarged for viewing individual clones. Count, frequency and diversity panels correspond to the read count, frequency (both non-symmetric) and the total number of clonotypes that are shared between samples. Pairwise overlaps are stacked, i.e., segment arc length is not equal to sample size.

Figure S3 Enlargement of the post-immunization transcriptional landscape of HSPCs B cell clonal repertoires. Pairwise overlap circos plots of HSPC B cell clonal repertoires (n=6mice/group) prepared using MiXCR software and shown in Figure 5A were enlarged for viewing individual clones. Count, frequency and diversity panels correspond to the read count, frequency (both non-symmetric) and the total number of clonotypes that are shared between samples. Pairwise overlaps are stacked, i.e., segment arc length is not equal to sample size.

Figure S4 Enlargement of the post-infection transcriptional landscape of HSPCs B cell clonal repertoires. Pairwise overlap circos plots of HSPC B cell clonal repertoires (n = 6mice/group) prepared using MiXCR software and shown in Figure 5A were enlarged for viewing individual clones. Count, frequency and diversity panels correspond to the read count, frequency (both non-symmetric) and the total number of clonotypes that are shared between samples. Pairwise overlaps are stacked, i.e., segment arc length is not equal to sample size.

Figure S5 Vaccine content determines gene set enrichment of HSPCs. RNAseq was performed on HSPCs isolated from CD-1 mice on days 1 and 3 post immunization with PBS, ACV, or WCV and on days 1 and 3 post subsequent infection with Bp. **(A)** Venn diagram was prepared for significant differentially expressed genes in HSPCs of ACV- and WCV-immunized mice when compared to PBS control mice. **(B)** Gene signatures enriched (fold change>5) in the WCV-immunized HSPC gene set are shown. **(C)** A Venn diagram was prepared for significant differential gene expression in HSPCs from PBS-, ACV-, and WCV-immunized and subsequently Bp challenged mice when compared to PBS control mice. **(D)**. Gene signatures enriched (fold change>5) in the PBS vaccinated, Bp challenged HSPCs gene set are shown. **(E)** HSPC gene signatures enriched (fold change>5) that overlap PBS vaccinated, Bp challenged and WCV-immunized, Bp challenged mice are shown. **(F)**. HSPC gene signatures enriched (fold change > 5) that overlap all Bp challenged mice are shown. Venn diagrams and gene set enrichment were established using Venny 2.1 and PANTHER, respectively. Significant data was determined by FDR (<0.05).

Table S1 Compositions of vaccines of this study.

Table S2 Flow cytometry antibodies used in this study.

Table S3 Summary of RNAseq performed in this study.
