# Supplementary figures and images for: Bordetella pertussis Whole Cell Immunization, Unlike Acellular Immunization, Mimics Naïve Infection by Driving Hematopoietic Stem and Progenitor Cell Expansion in Mice
Source: Front Immunol. 2018 Oct 18;9:2376. doi: 10.3389/fimmu.2018.02376 (PMC6200895; doi:10.3389/fimmu.2018.02376)

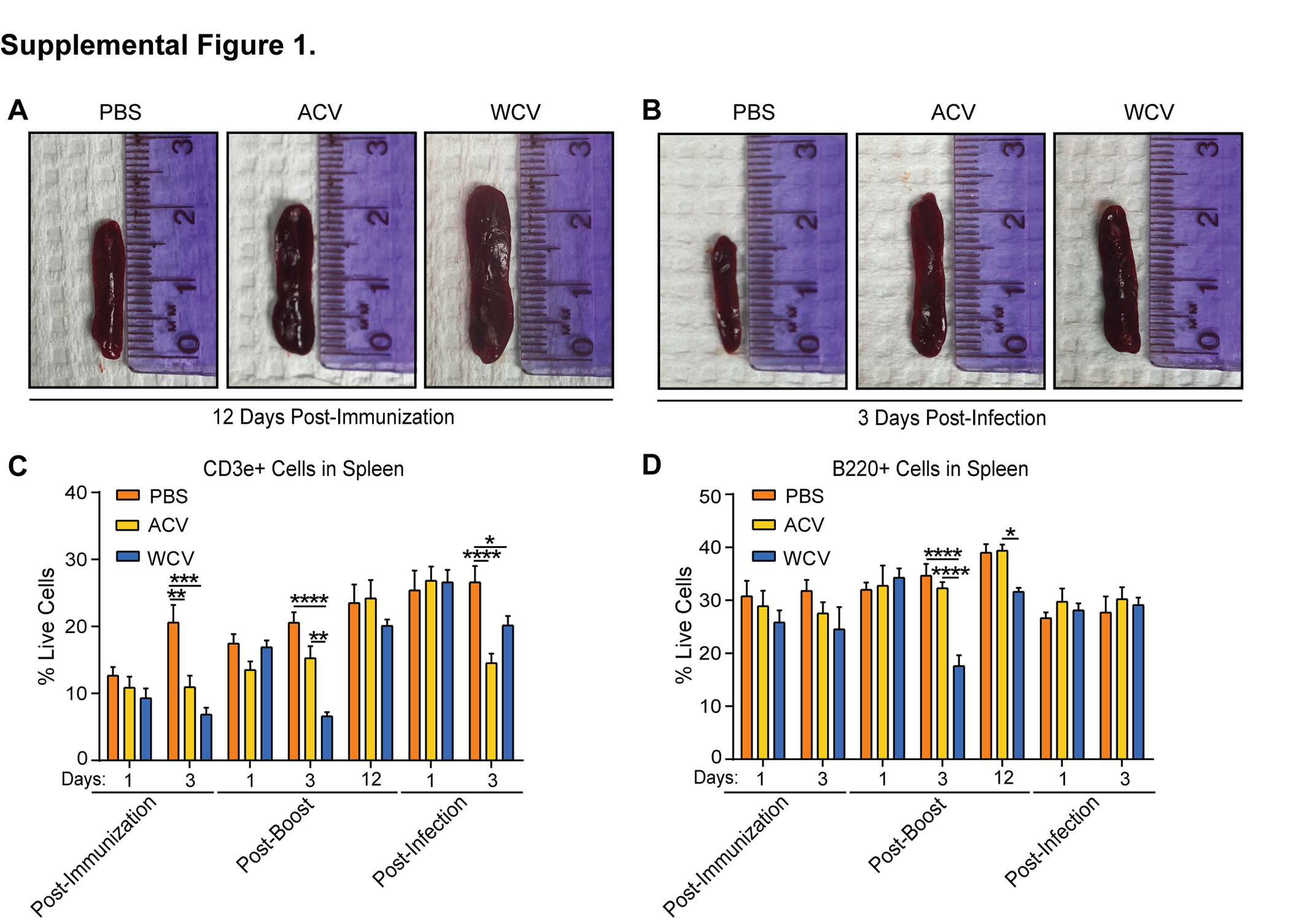

Supplement: Figure S1 — WCV induces alterations in spleen size and B and T cell population composition. (A). Spleen sizes from CD-1 mice (n = 4/group for each time point) immunized with phosphate buffered saline (PBS), Bp acellular vaccine (ACV), or Bp whole cell vaccine (WCV) are shown in representative images for the indicated time points across the immunization and infection schedule. (B). Representative dot plots from flow cytometric analysis described in Figure 2B. (C). Total CD3e+ cells in the spleen (n = 4/group for each time point) were calculated by multiplying the percentage of CD3e+ live cells (Figure 2C) in the spleen by total number of live cells in the spleen. (D) Total B220+ cells in the spleen (n = 4/group for each time point) were calculated by multiplying the percentage of B220+ live cells in the spleen (Figure 2D) by total number of live cells in the spleen. *p < 0.05; **p < 0.01; ***p < 0.001; ****p < 0.0001; 2-way ANOVAs with Tukey's multiple comparisons. Error bars are mean ± SEM values. [file Image_1.TIF]

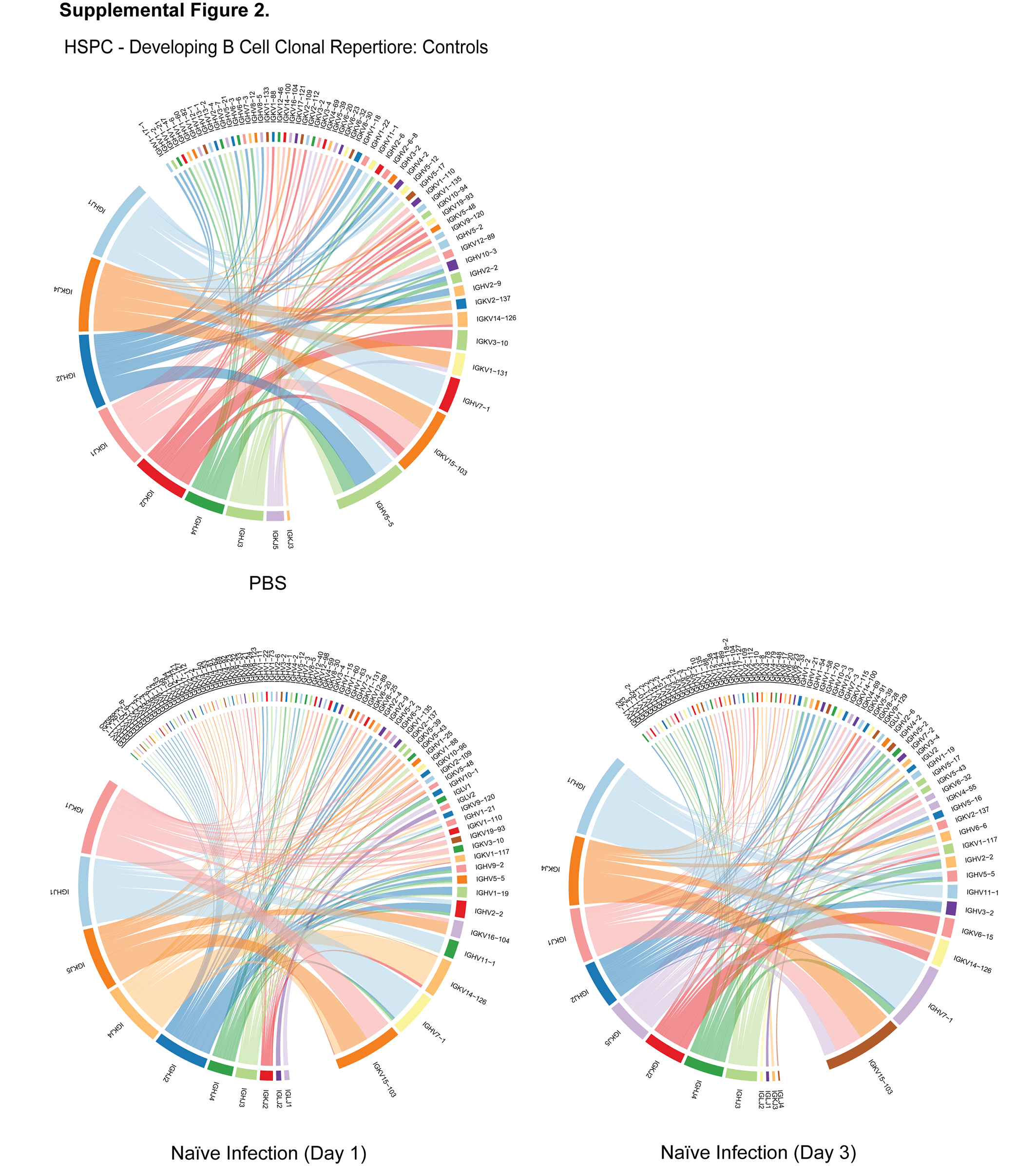

Supplement: Figure S2 — Enlargement of controls for the transcriptional landscape of HSPCs B cell clonal repertoires. Pairwise overlap circos plots of HSPC B cell clonal repertoires (n = 6mice/group) prepared using MiXCR software and shown in Figure 5A were enlarged for viewing individual clones. Count, frequency and diversity panels correspond to the read count, frequency (both non-symmetric) and the total number of clonotypes that are shared between samples. Pairwise overlaps are stacked, i.e., segment arc length is not equal to sample size. [file Image_2.TIF]

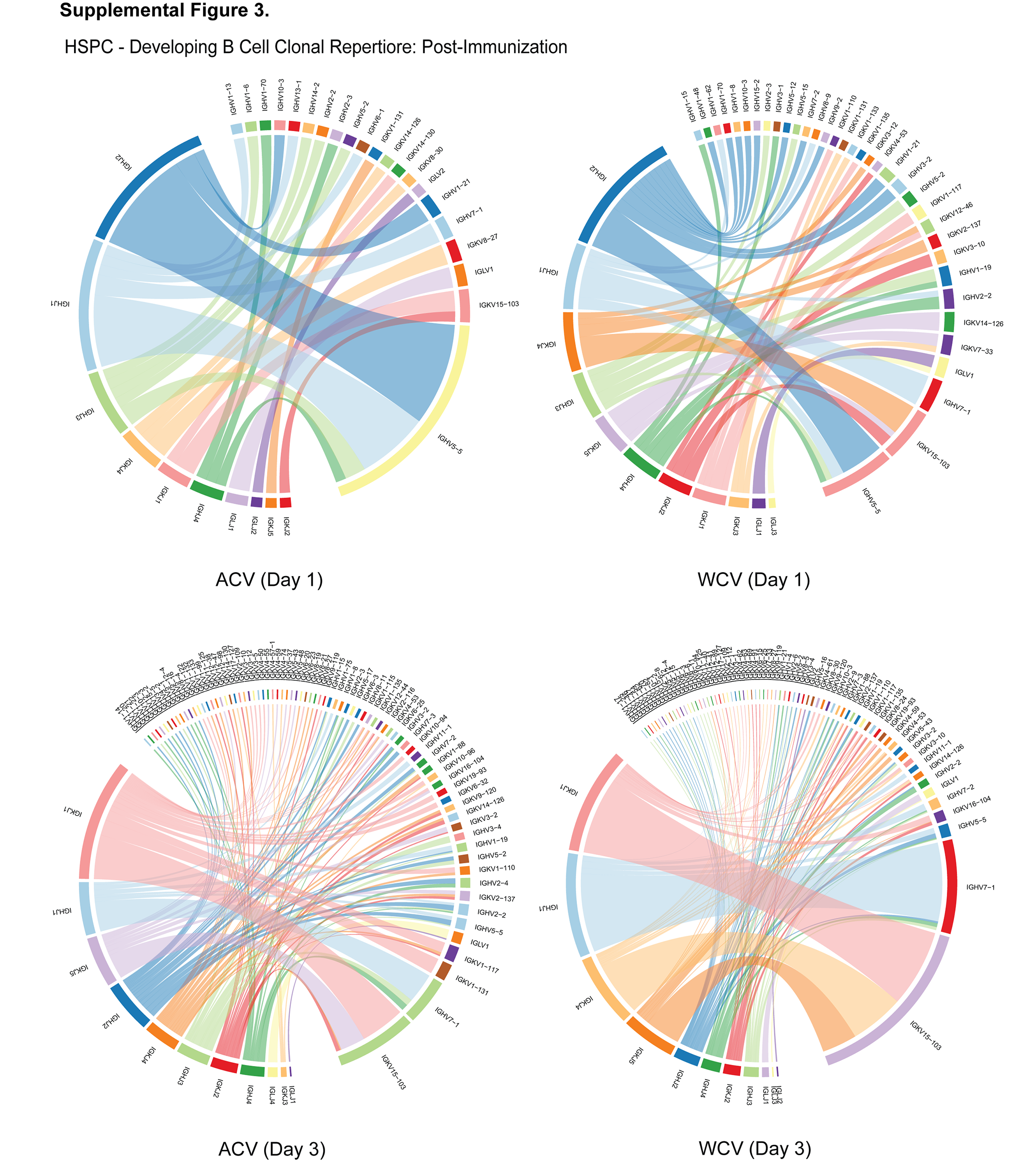

Supplement: Figure S3 — Enlargement of the post-immunization transcriptional landscape of HSPCs B cell clonal repertoires. Pairwise overlap circos plots of HSPC B cell clonal repertoires (n=6mice/group) prepared using MiXCR software and shown in Figure 5A were enlarged for viewing individual clones. Count, frequency and diversity panels correspond to the read count, frequency (both non-symmetric) and the total number of clonotypes that are shared between samples. Pairwise overlaps are stacked, i.e., segment arc length is not equal to sample size. [file Image_3.TIF]

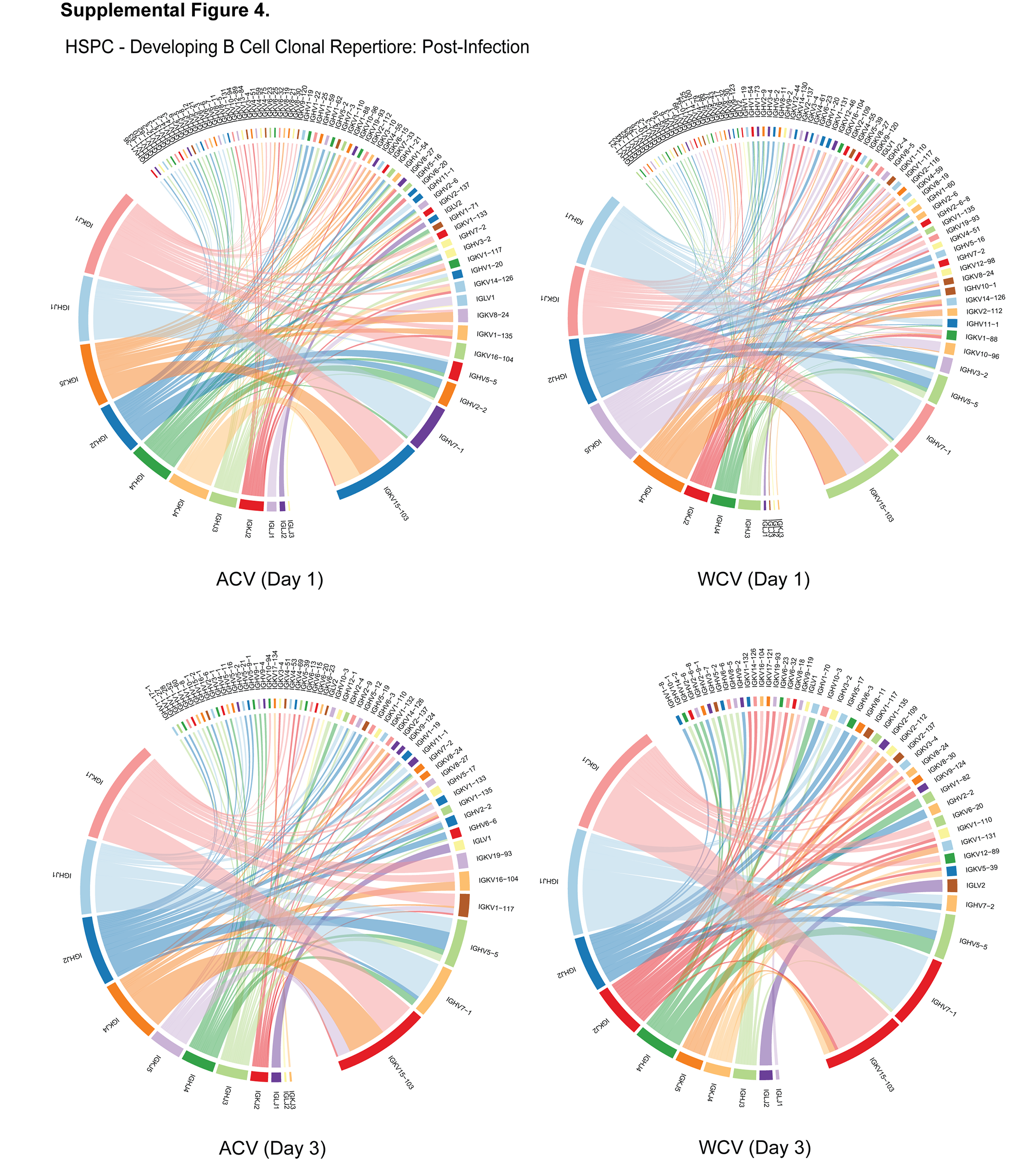

Supplement: Figure S4 — Enlargement of the post-infection transcriptional landscape of HSPCs B cell clonal repertoires. Pairwise overlap circos plots of HSPC B cell clonal repertoires (n = 6mice/group) prepared using MiXCR software and shown in Figure 5A were enlarged for viewing individual clones. Count, frequency and diversity panels correspond to the read count, frequency (both non-symmetric) and the total number of clonotypes that are shared between samples. Pairwise overlaps are stacked, i.e., segment arc length is not equal to sample size. [file Image_4.TIF]

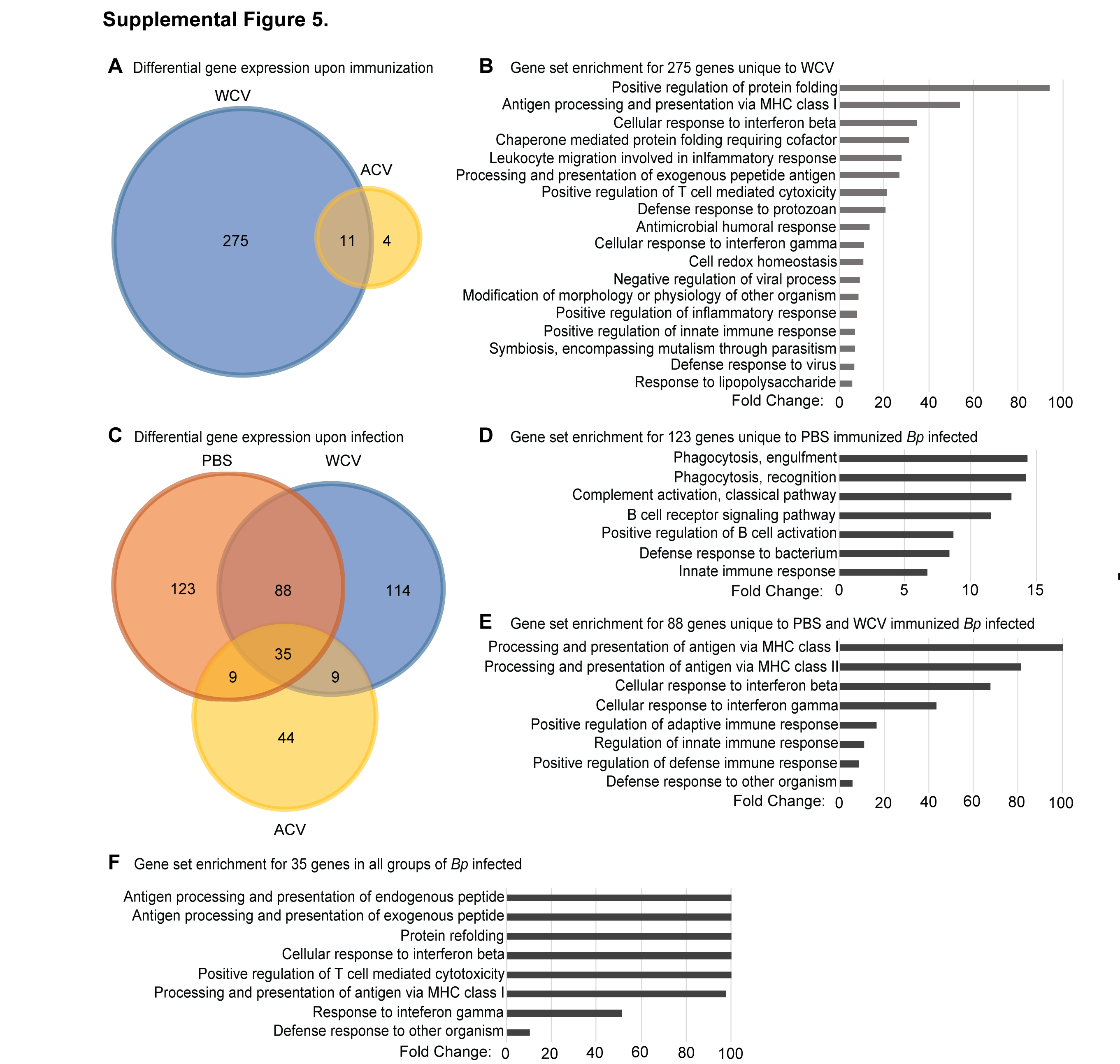

Supplement: Figure S5 — Vaccine content determines gene set enrichment of HSPCs. RNAseq was performed on HSPCs isolated from CD-1 mice on days 1 and 3 post immunization with PBS, ACV, or WCV and on days 1 and 3 post subsequent infection with Bp. (A) Venn diagram was prepared for significant differentially expressed genes in HSPCs of ACV- and WCV-immunized mice when compared to PBS control mice. (B) Gene signatures enriched (fold change>5) in the WCV-immunized HSPC gene set are shown. (C) A Venn diagram was prepared for significant differential gene expression in HSPCs from PBS-, ACV-, and WCV-immunized and subsequently Bp challenged mice when compared to PBS control mice. (D). Gene signatures enriched (fold change>5) in the PBS vaccinated, Bp challenged HSPCs gene set are shown. (E) HSPC gene signatures enriched (fold change>5) that overlap PBS vaccinated, Bp challenged and WCV-immunized, Bp challenged mice are shown. (F). HSPC gene signatures enriched (fold change > 5) that overlap all Bp challenged mice are shown. Venn diagrams and gene set enrichment were established using Venny 2.1 and PANTHER, respectively. Significant data was determined by FDR (<0.05). [file Image_5.TIF]
