# Supplementary material for: Bordetella pertussis Whole Cell Immunization, Unlike Acellular Immunization, Mimics Naïve Infection by Driving Hematopoietic Stem and Progenitor Cell Expansion in Mice
Source: Front Immunol. 2018 Oct 18;9:2376. doi: 10.3389/fimmu.2018.02376 (PMC6200895; doi:10.3389/fimmu.2018.02376)
Supplement: Table S1 — Compositions of vaccines of this study. [file Table_1.pdf]

Table S1. Compositions of vaccines of this study.

	Vaccine groups	
**Vaccine component***	**ACV (1/5th human dose)**	**WCV**(1/5th human dose)**
Pertussis Toxoid***	5	0.4
Filamentous Hemagglutinin	5	3.5
Pertactin	1.6	0.3
Adenylate Cyclase Toxin	0	0.2
Aluminum hydroxide	125	0
Other antigens/adjuvants	0	62

***All masses of antigens or adjuvant are indicated in µg

** This estimate is based on the number of peptides identified by mass for each antigen in the WCV. The percentage is then used to estimate the potential mass based on the fact that the WCV dose used in this study contained 66 µg of total protein.

*** For total pertussis toxin, the sum of PtxA,B,C,D peptides was combined.
