# Supplementary material for: Bordetella pertussis Whole Cell Immunization, Unlike Acellular Immunization, Mimics Naïve Infection by Driving Hematopoietic Stem and Progenitor Cell Expansion in Mice
Source: Front Immunol. 2018 Oct 18;9:2376. doi: 10.3389/fimmu.2018.02376 (PMC6200895; doi:10.3389/fimmu.2018.02376)
Supplement: Table S2 — Flow cytometry antibodies used in this study. [file Data_Sheet_2.PDF]

**Table S2. Flow cytometry antibodies used in this study.**

| Antibody | Fluorphore | Clone | Catalog Number |
|---|---|---|---|
| CD117 | APC-R700 | 2B8 | 565476 |
| CD11b | BB515 | M1/70 | 564454 |
| CD127 | PE-CF594 | SB/199 | 562419 |
| CD150 | BV421 | Q38-480 | 562811 |
| CD16/CD32 | APC-Cy™7 | 2.4G2 | 560541 |
| CD24 | BV421 | M1/69 | 562563 |
| CD34 | Alexa Fluor® 647 | RAM34 | 560230 |
| CD3e | BV510 | 145-2C11 | 563024 |
| CD43 | BB515 | S7 | 564646 |
| CD45R/B220 | APC-Cy™7 | RA3-6B2 | 552094 |
| CD48 | PE | HM48-1 | 557485 |
| IgD | APC | 11-26c.2a | 560868 |
| IgM | PE-CF594 | R6-60.2 | 562565 |
| Lineage Antibody Cocktail, with Isotype Control | PerCP-Cy™5.5 | | 561317 |
| Ly-6A/E | BB515 | D7 | 565397 |
| Ly-6G and Ly-6C | PE | RB6-8C5 | 553128 |
| Lγ-51 (BP-1) | PE | BP-1 | 553735 |

All antibodies used in this study were purchased from BD Biosciences.
